# Imagining Change: An Integrative Approach toward Explaining the Motivational Role of Mental Imagery in Pro-environmental Behavior

**DOI:** 10.3389/fpsyg.2016.01780

**Published:** 2016-11-17

**Authors:** Christine Boomsma, Sabine Pahl, Jackie Andrade

**Affiliations:** School of Psychology, Plymouth UniversityPlymouth, UK

**Keywords:** mental images, visual communication, pro-environmental goals, behavior change, environmental change

## Abstract

Climate change and other long-term environmental issues are often perceived as abstract and difficult to imagine. The images a person associates with environmental change, i.e., a person’s environmental mental images, can be influenced by the visual information they come across in the public domain. This paper reviews the literature on this topic across social, environmental, and cognitive psychology, and the wider social sciences; thereby responding to a call for more critical investigations into people’s responses to visual information. By integrating the literature we come to a better understanding of the lack in vivid and concrete environmental mental imagery reported by the public, the link between environmental mental images and goals, and how affectively charged external images could help in making mental imagery less abstract. Preliminary research reports on the development of a new measure of environmental mental imagery and three tests of the relationship between environmental mental imagery, pro-environmental goals and behavior. Furthermore, the paper provides a program of research, drawing upon approaches from different disciplines, to set out the next steps needed to examine how and why we should encourage the public to imagine environmental change.

## Introduction

Images play an important role in psychological processes such as memory and behavioral planning. This paper will review research into the images that individuals associate with climate change and other long-term environmental changes (from here on referred to as environmental change). We will use the term environmental mental imagery when referring to these personal images, defined as mental representations which take the form of sensory images (e.g., visual images, sounds and emotions; Leiserowitz, 2006; Andrade et al., 2012). Environmental mental imagery may be formed in different ways, for example through personal experiences or personal imagination; this paper focuses on how these images are influenced by the external environmental images that are available in the public domain. This paper is structured around two main questions. First, what is the effect of external images – such as those in the media – on people’s environmental mental imagery? And second, how does being able to vividly imagine environmental change influence people’s pro-environmental behavior?

Traditionally, environmental psychology has relied mostly on social psychology approaches, and less on cognitive psychology perspectives – but integrative psychology research is needed to tackle the environmental challenges we face today (Clayton et al., 2016). The main aim of this paper is to provide a novel integrative approach to studying environmental mental imagery building on research in environmental psychology, social psychology, cognitive psychology and environmental social science, and working toward a program of research set out at the end of this paper. The paper also presents preliminary data from three studies that combine these lines of research, exploring some of the relationships brought forward.

## Literature Review

### The Invisibility of Environmental Change

Most people have at least some knowledge about the causes and effects of climate change, but this knowledge does not necessarily lead to major and necessary changes in behavior (Lorenzoni et al., 2007). And, even though only a minority completely rejects climate change, it is becoming more common to doubt the reality and severity of climate change (Whitmarsh, 2011). Research has identified several common social and individual barriers (Lorenzoni et al., 2007; Gifford, 2011 for an overview) that prevent individuals from engaging with environmental change. Many of these barriers are linked to the fact that environmental change is a complex, long-term, and distant issue (Lorenzoni et al., 2007).

Environmental issues tend to have a slow onset and consequences are mostly long-term (Moser and Dilling, 2004). This makes it difficult for individuals to understand how their behavior at the moment can affect the future, and to relate their individual actions to the larger context of environmental change (Tickell, 2002; Sheppard, 2005; Pahl et al., 2014). Also, environmental change is often associated with problems that are happening far away (from a European perspective), such as ice-caps melting in the artic, loss of biodiversity in the Great Barrier Reef, and plastic pollution in the Pacific Ocean. Consequently, people tend to perceive environmental change as something that is threatening plants, animals, and people in other parts of the world. Individuals have difficulty imagining how these problems that are happening far away can have an effect on themselves locally (Gardner and Stern, 2002; Leiserowitz, 2005, 2006). To help overcome this sense of invisibility scholars have suggested that communicators need to use clear images to educate the public about environmental change and to motivate them to change behavior (Moser, 2010). As Sheppard (2005) puts it: seeing believes. Next, the paper will discuss how images are used in the public domain. We summarize the studies which have started to explore the relationship between these publically available images of environmental change and environmental mental imagery. Some important research has been done, focusing mainly on describing existing environmental mental imagery in the public using a qualitative approach. Following this overview, the paper will discuss a large body of research within cognitive psychology on mental imagery and the formation of goals. By integrating these fields of research, important parallels between findings from different disciplines can be drawn which adds considerably to our knowledge regarding environmental mental imagery and its effect on behavior, and opens up new avenues of research.

### Current Use of Environmental Change Imagery in the Public Domain

Visual information is often used in the media, by newspapers, television, film, communications by NGOs and others, to illustrate environmental change and also to motivate the public to behave more sustainably (see O’Neill and Smith, 2014 for an overview). In a review of climate change imagery used in British broadsheet and tabloid newspaper coverage of climate change, Smith and Joffe (2009) found that nearly two thirds of articles sampled were accompanied by an image. Images that accompany climate change articles are not chosen indiscriminately. A comprehensive review of the use of climate change imagery in the media (i.e., UK, US, and Australian online newspapers) showed that visual imagery is often used to frame climate change as a ‘contested’ or ‘distant’ issue; although the dominant visual frame varies between countries and depends on newspaper ownership (O’Neill, 2013). Visuals tend to take the form of ‘global imagery’: abstract and decontextualized (Hansen and Machin, 2013). That is, images do not necessarily directly visualize the article topic, nor do they place the topic in a local context. In a review of images included alongside articles in Canadian newspapers, DiFrancesco and Young (2010, p. 525) conclude that “rather than being complementary, many image – language combinations appear to be dissociated and pulling the narrative in different directions.” Newspapers aren’t the only source of environmental change images; there is a wide range of other sources of environmental change images available in the public domain. For instance, scholars have examined people’s environmental beliefs and perceptions in response to portrayals of climate change in movies (cf. Leiserowitz, 2004; Lowe et al., 2006). Furthermore, visual information plays an important role in media appeals by environmental interest groups (Nicholson-Cole, 2005; Doyle, 2007).

Given the benefits of visual information in engaging individuals it is perhaps not surprising that visuals are so widely used in the media. Visuals can show people something that is normally not (directly) visible. Pictorial warnings on cigarette packages which depict the (invisible) health consequences of smoking have repeatedly been shown to be more effective in reducing intentions to smoke compared to textual warnings (see Hammond, 2011 for a review). Based on examples of the use of visuals in the public domain, Tufte (1990) argues that visual displays are an appropriate, and often even optimal, way to present information when taking into account human cognitive capabilities. Moreover, images can evoke strong affective responses (Smith and Joffe, 2009), easily link to emotions (Holmes and Mathews, 2005, 2010; Sheppard, 2005), and goals (Conway et al., 2004). Furthermore, visual information is able to concretise risks (Smith and Joffe, 2009), condense complex information, convey strong messages, and provide the basis for personal thoughts and conversations (Nicholson-Cole, 2005).

### Effect of Visuals on Personal Environmental Mental Imagery

Little is known about how this commonly used and widely available visual information in the public domain affects the way people visualize environmental change themselves, and thus the environmental mental images that people form. In the environmental context, studies on responses to visual information are limited (Moser, 2010). Scholars have noted that external imagery is key in determining how individuals perceive real-world issues and can transform abstract issues into something that can be visualized – and that individuals can respond to (O’Neill et al., 2013). As noted by Smith and Joffe (2009): “visual information is likely to play a powerful role in positioning public conceptions of climate change.” O’Neill (2013, p. 18) takes this statement further by noting that some types of visuals can “come to ‘stand in’ for a multitude of climate change meanings.” Many authors in the environmental domain have argued that we need to critically explore responses to visual information, and how people engage with visual representations (Sheppard, 2001, 2005; Nicholson-Cole, 2005; Jude, 2008; Anderson, 2009; Moser, 2010; Hansen and Machin, 2013; O’Neill, 2013; O’Neill et al., 2013; O’Neill and Smith, 2014).

One area of research that explores the relationship between visual information in the public domain and people’s environmental mental imagery is research into the public’s image associations with climate change. In a national representative survey of the US public in 2002, Leiserowitz (2006) found that the current and projected impact of climate change (e.g., melting glaciers) were the most salient images in the public, and these images were mostly associated with negative feelings. Cross-national research showed that the majority of associations with climate change lack personal relevance and focus on impacts rather than causes and solutions (Lorenzoni et al., 2006). Scholars have argued that environmental mental imagery can ‘mirror’ representations of environmental change commonly used in the media (Joffe, 2008; Smith and Joffe, 2009). Research by Nicholson-Cole (2005) further suggests that the way participants conceptualize climate change visually is particularly linked to visual information appearing in the media. Following 30 semi-structured interviews the author reported that participants found it difficult to imagine the future. Following the often catastrophic imagery used in the media, the imagery that was described by the participants tended to be negative in nature (though positive imagery was mentioned as well).

Thus, the visual information on environmental change that individuals are exposed to in the public domain is an important factor for determining the environmental mental images that people form. But, how do these environmental mental images influence people’s pro-environmental behavior? Some researchers have started to explore the relationship between imagery and behavior, but this area of research is still in its infancy (Lorenzoni et al., 2006). Recent findings provide an indication of the effect that environmental imagery could have on climate change beliefs. It has been suggested that affective imagery could be one of the strongest predictors of climate change risk perception and policy preferences, compared to cultural worldviews, socio demographic and political variables (Leiserowitz, 2006; Smith and Leiserowitz, 2012). So, it seems that people’s climate change perceptions and attitudes are related to their mental imagery on the issue. However, the frequent use of global and abstract imagery in the media may prevent people from seeing climate change as a personally relevant issue that influences their behavior and policy preferences (Nicholson-Cole, 2005; Leiserowitz, 2006). This need for a personal connection or relevance is illustrated in a study by Hershfield et al. (2014); in this study individuals were more willing to engage in pro-environmental behavior if they thought their country had a long future and they felt a close connection to future generations. One theoretical model which attempts to explain what information individuals draw on to mentally represent environmental change is Construal Level Theory (Trope and Liberman, 2003; Liberman and Trope, 2008). The theory proposes that events perceived as distant (i.e., temporal, spatial, social and/or in likelihood of occurance), are represented in abstract and decontextualized terms, while less distant events are represented using rich detail of the situation (Trope and Liberman, 2003; Liberman and Trope, 2008). So, according to Construal Level Theory people form concrete or more abstract representations of an event if they think of the event as close or distant. In a recent study, Leviston et al. (2014) found that individuals who reported greater acceptance of climate change also tended to report more concrete environmental mental images. However, the authors note that there is some uncertainty about the direction of this effect. Perhaps we can draw upon research from other domains to further explain how environmental mental imagery may be linked to pro-environmental behavior. The role of mental imagery as a key motivator in shaping attitudes and behavior has been acknowledged by research in cognitive psychology for other areas (not pro-environmental behaviors). To our knowledge, these lines of research have not been linked in the literature to date. Reviewing the cognitive literature can provide us with important insights on the underlying mechanisms that can explain the relationship between mental imagery and pro-environmental behavior.

### The Relationship between Mental Imagery and Goals: Insights from Cognitive Psychology

Research in cognitive psychology suggests that part of the motivational influence of mental imagery results from its link to goals. According to Conway et al. (2004) goals cannot be assessed directly, but they can be understood from looking at the representations derived from them in the form of emotions, verbal statements, actions, and most importantly mental images. So, mental images can represent information about goals. Goals, also referred to as goal intentions (Achtziger et al., 2008), direct action by determining what people attend to, what knowledge and attitudes are most accessible and how people evaluate a situation (Lindenberg and Steg, 2007). Locke and Latham (1990) describe them as stable cognitive representations of motivational impulses. Goals prompt and guide complex behaviors over time (Lindenberg and Steg, 2007).

A cognitive theory which takes mental imagery and goals as core concepts is the Elaborated Intrusion Theory of Human Motivation (EI Theory; Kavanagh et al., 2005). EI Theory assumes that goals are triggered by internal or external triggers (e.g., external cues, associated thoughts, and physiological deficits) and experienced in the form of seemingly spontaneous intrusive thoughts. These thoughts can take the form of verbal or visual fragments (May et al., 2004; Kavanagh et al., 2005), and are intrusive in the sense that they can interrupt day to day activity and can be relatively difficult to control (Brewin et al., 1996). Intrusive thoughts may relate to immediate goals (‘I need a drink’), long-term goals (‘I need to get fit’), or value-related goals (‘I need to recycle this’). But, this process alone lacks motivational impact; more conscious, controlled processes are needed to sustain motivation. An essential part of this elaboration process is the formation of sensory mental images: it is thought that imagery-based elaboration of intrusive thoughts can sustain motivation (Andrade et al., 2012). The motivational power of mental imagery is strengthened through its close link with emotions. Mental images can easily access emotional episodes from the past, and they can reactivate these emotions and feelings, enabling an individual to relive the emotion (Holmes and Mathews, 2010). They also connect to emotions by their ability to simulate reality. When mental imagery takes place, brain areas are activated that are involved in processing the imagined event, action or information in reality (cf. Ganis et al., 2004; Cabeza and St Jacques, 2007; Kim et al., 2007; Sharot et al., 2007). Holmes and Mathews (2010, p. 352) conclude that: “images are interpreted as being similar to real emotion-arousing events due to overlapping activation patterns between imagery and perception.”

The motivational impact of mental imagery, following the EI Theory framework, has been supported for food, drink and tobacco cravings (May et al., 2004, 2008; Kavanagh et al., 2009; Connor et al., 2014), and the desire to play sport (May et al., 2008). In these studies a higher imagery frequency was associated with strength of desire, and with more frequent behavior (e.g., Connor et al., 2014). Holmes and Mathews (2010) also mention the importance of mental images in guiding behavior. Imagining a possible event outcome increases our belief that this outcome will occur, and imagining our own future behavior increases the chances of enacting this behavior. In sum, mental images maintain and reflect information about goals. An implication from this is that imagery can increase readiness for action and can promote enacting on imagined behavior (Holmes and Mathews, 2010). This is in line with the reasoning that mental images not only guide and maintain behavior, as discussed above, but can also initiate behavior change. The goal system has been shown to be reluctant to change but, by generating new mental images, new goals might be formed (Conway et al., 2004). Mental images help individuals choose between different goals; i.e. mental images can help make a goal more focal, and increase its influence on behavior. intriguingly, this suggests that the imagineability of a goal is important and raises the possibility that it is harder to achieve goals that are more difficult to imagine.

### Applying a Cognitive Framework to Environmental Mental Imagery

Moving to the environmental context, it can be argued that the observed relationship between environmental mental imagery, pro-environmental beliefs and behavior (in Leiserowitz, 2006; Smith and Leiserowitz, 2012) may follow from its strong link to the goal system. This line of reasoning can be strengthened by drawing on further research from cognitive psychology to explain why many people currently lack vivid, concrete images of environmental change. Generalized goals to change our lives (e.g., living healthier/adopting a pro-environmental lifestyle) are less vividly imagined because they are often more abstract and distant in time, as well as being less familiar. For these generalized, long-term goals, there is less information available in memory to draw on, as a result imagery focusing on distant consequences will have less emotive power (Andrade et al., 2012). Short-term goals involve more familiar behaviors that are more richly encoded in memory and can therefore be imagined more vividly, which increases their motivational power (May et al., 2008; Kavanagh et al., 2009). Abstract information about environmental change might be difficult to retrieve from memory so the related risks are underestimated (Slovic et al., 2004; Weber, 2006), and the sensory information needed to form vivid, motivating images is lacking. Long-term goals can be made less abstract by forming implementation intentions, or specific behavioral plans, thus increasing the cognitive availability of long-term goals (Gollwitzer and Sheeran, 2006; Andrade et al., 2012). Providing concrete information about specific future events (e.g., specific impacts of environmental change on a familiar place; Sheppard, 2005) will increase the value attached to those future events (Kim et al., 2013). It is expected that external images can motivate behavior by similar mechanisms. By internalizing external environmental change images, a shift could be made toward long-term pro-environmental goals.

So, strong affectively charged external environmental change images could be internalized as environmental mental images to: (1) increase the motivational power of (new or existing) pro-environmental goals which guide and maintain behavior; and (2) serve as triggers for, or initiate, pro-environmental behavior. It is important to note here that elaboration and the formation of mental imagery are particularly likely when the topic has affective meaning for the individual (May et al., 2004; Kavanagh et al., 2005). Whether an image has affective meaning and can be internalized successfully is likely to depend on the characteristics of the message (e.g., image design, message framing) and the characteristics of the message recipient (e.g., individual values, beliefs, previous knowledge). This rationale provides further opportunities for the integration between the study of environmental mental imagery and other lines of research in psychology. For instance, the proposed relationship between elaboration and affective meaning seems to link to research suggesting that individuals will elaborate more on environmental messages when they see protecting the environment as an important value in life (Schwartz, 1992, 1994; Lorenzoni et al., 2007; Steg et al., 2012; Boomsma and Steg, 2014; see Experience of Environmental Mental Imagery). Furthermore, given the strong relationship between mental images and emotions, of particular relevance with regards to message design is research on emotional message framing (e.g., the use of fear; see Experience of Environmental Mental Imagery).

### Summary

As mentioned in Section “Effect of Visuals on Personal Environmental Mental Imagery” research on responses to visual information and the effect of environmental mental imagery is limited. We have discussed two lines of research that have explored these issues to some extent. Research from environmental psychology and social science shows that the environmental change imagery available in the public domain influences people’s environmental mental imagery. These mental images in turn have been shown to be related to perceived issue relevance, policy preferences, and climate change acceptance. Cognitive psychology has demonstrated a strong link between mental imagery and goals, with mental imagery being strongly connected to motivational and emotional pathways. In the next section, we will present three small studies that examine how specific environmental visuals are internalized by individuals using quantitative, and some qualitative, data. These studies are a first step in a research program outlined in the final sections of this paper. They build on the research on mental imagery in social science and cognitive psychology, introduce a new scale to measure environmental mental imagery, and start to explore the underlying mechanisms by which environmental mental imagery could influence pro-environmental behavior. Specifically, these studies address the question of whether individuals experience environmental mental imagery after exposure to a visual pro-environmental message and, if they do, how is that mental imagery related to pro-environmental goals and behavior change?

## Study A

### Materials and Methods

#### Participants and Procedure

In Study A, 16 university students (enrolled on a module on psychology and sustainability) saw a 7 min video on marine plastic pollution during a lecture. For a detailed description of the content of the video see Appendix A (full materials are available online, doi: 10.13140/RG.2.2.12718.92484). During a subsequent lecture, 5 weeks after seeing the video, survey data was collected. Participants were told that the survey would measure what they remembered about the message they had seen previously, and what had happened since they saw the message. In addition, they were told that their answers would be stored anonymously and that they had the right to withdraw at any time. Participants only filled in the survey if they had given verbal consent to participate in the study. After filling in the survey participants were debriefed verbally and any remaining questions were answered.

#### Measures

An *Environmental Mental Imagery* scale was developed based on a questionnaire by Kavanagh et al. (2009). The scale used in Study A covered mental imagery of the message itself and of related problems. Two items were included to measure strength (While thinking about the message – how vividly did you recall images from the video; how vividly did you imagine problems related to the plastic debris floating in the oceans), and two items were included to measure frequency (How often – did you recall images from the message; did you imagine problems related to the plastic debris floating in the oceans) of mental imagery related to the message. Participants were asked to rate what happened at the time they thought back to the video on a scale ranging from 1 (Not at all) to 11 (Extremely or Constantly, for the strength and frequency items respectively). A mean score was calculated for environmental mental imagery (four items, Cronbach’s α = 0.74).

*Content of environmental mental imagery* was assessed with an open-ended question similar to that used by Leiserowitz (2006). Participants were asked to describe the picture that had stuck in their mind most when they thought back to the message. In addition, two items were included to ask participants about the extent they thought about the images and the text when they thought back to the message. The ‘image’ item was: “When you thought back to the message to what extent did you think about the images,” with a response scale ranging from 1 (Not at all) to 5 (I only thought about the images). The ‘text’ item referred to the verbal content of the video: “When you thought back to the message to what extent did you think about the explanation the expert gave for the garbage patch.” The response scale ranged from 1 (Not at all), to 5 (I only thought about the explanation of the captain).

The formation of *pro-environmental goals* was measured as pro-environmental thoughts. Participants were asked: “When you thought back to the message to what extent did you think about what you could do in your day-to-day life to prevent the problem from getting worse.” Responses were indicated on a scale ranging from 1 (Not at all) to 5 (I only thought about what I could do).

### Results

#### Experience of Environmental Mental Imagery

Mean scores on mental imagery for all three studies are reported in **Table 1**. In Study A, participants indicated experiencing quite vivid environmental mental imagery related to the video, even after 5 weeks. Participants reported thinking back to the visual content of the message to a larger extent compared to the verbal content of the message, *t*(15) = 4.21, *p* = 0.001, *d* = 1.07. A content analysis was conducted on the responses to the open question, in line with the previous finding the majority (*N* = 12) of the descriptions was based on the visual content of the video – the remaining descriptions were based on a combination of verbal and visual content from the video. Furthermore, the qualitative data summarized in **Table 2** revealed that, similar to previous studies, imagery descriptions took the form of simple word associations or short narrative statements (cf. Smith and Leiserowitz, 2012).

**Table 1:** Mean scores on environmental mental imagery, thoughts about the visual and verbal content and environmental values for studies A, B, and C.

	*M(SD)* environmental mental imagery^∗^	*M(SD)* thoughts about visual content^∗∗^	*M(SD)* thoughts about verbal content^∗∗^	*M(SD)* environmental values^∗∗∗^
Study A	6.82 (1.60)	3.94 (0.93)	2.50 (1.21)	
Study B	5.40 (1.90)	2.53 (1.30)	2.12 (1.03)	4.95 (1.47)
Study C	5.25 (2.00)	2.45 (1.16)	2.13 (1.05)	4.24 (1.31)

*^∗^Response scale environmental mental imagery 1 (Not at all) – 11 (Extremely/Constantly). ^∗∗^Response scale visual and verbal content 1 (Not at all) – 5 (I only thought about …). ^∗∗∗^Response scale environmental values -1 (opposed to the values), 0 (not at all important) - 7 (of supreme importance).*

**Table 2:** Examples of responses to the question ‘Please describe the picture that has stuck in your mind most when you think back to the message now..’ for Studies A, B, and C.

	Simple word associations	Short narrative statements
Study A	“*Vast amount of waste in the ocean*”	*“Plastic bags and rubbish lurking in the ocean, being picked up. The sheer amount of rubbish in the ocean, what potential problems this will cause, beaches with rubbish, plastic bags on”*
Study B	“*Wall insulation*”	*“The picture of energy escaping mostly in the doors and windows if you did not have [double] glazing, also the loft insulation”*
Study C	“*The flooding*”	*“The ‘community’ offices where people work locally. It has large green spaces and there is a pond outside the building. The building is big but has many windows, it is modern and looks like it tries to bring the outside ‘green’ space into the building. The area looks clean and fresh”*

#### Relationship between Environmental Mental Imagery and Pro-environmental Goals

The correlations between environmental mental imagery and pro-environmental goals for all three studies are reported in **Table 3**. In Study A, pro-environmental goals, measured as pro-environmental thoughts, correlated with environmental mental imagery: participants who reported experiencing more vivid mental imagery, also reported more pro-environmental thoughts.

**Table 3:** Mean scores and correlations between behavior-related measures and environmental mental imagery for studies A, B, and C.

	Behavior-related measure^∗^	*M* (*SD*)	Correlation with environmental mental imagery
Study A	Pro-environmental goals (Thoughts)	3.06 (1.06)	*r* = 0.65, *p* = 0.009
Study B	Pro-environmental goals (Thoughts)	2.95 (1.11)	*r* = 0.44, *p* = 0.003
	Self-reported behavior change – energy related behaviors	3.59 (0.47)	*r* = 0.38, *p* = 0.024
Study C	Pro-environmental goals (Thoughts)	2.94 (1.14)	*r* = 0.51, *p* < 0.001
	Difficult goal intentions	3.53 (1.24)	*r* = 0.36, *p* = 0.001
	Easy goal intentions	5.76 (0.78)	*r* = 0.15, *p* = 0.192
	Self-reported behavior change – general sustainability behaviors	3.58 (0.50)	*r* = 0.27, *p* = 0.018

*^∗^Response scale: Pro-environmental goals 1 (Not at all) – 5 (I only thought about what I could do); Goal intentions 1 (Probably not) – 7 (Yes, definitely); Self-reported behaviour change 1 (Less) – 5 (More).*

## Study B

### Materials and Methods

#### Participants and Procedure

In Study B, 43 university students (11 men, mean age = 27.1, *SD* = 11.3) were recruited via the university participant pool in return for course credit. In a lab study participants were shown a slideshow (include images and text) on heat loss in the home, using images varying in vividness. There were two conditions: *N* = 22 received a vivid message with thermal images; *N* = 21 received a less-vivid message with schematic images, further described in Section “Experience of Environmental Mental Imagery” and Appendix A (full materials are available online, doi: 10.13140/RG.2.2.12718.92484). Participants returned to the lab 1 week later to fill in a survey. All participants signed an informed consent form before participating in the study and received a paper debrief with contact details upon finishing the study. The information they received about the survey beforehand was similar to Study A.

#### Measures

The measures from Study A were included again with some small adjustments. Two items were added to the *Environmental Mental Imagery scale* to measure mental imagery of future actions: “While thinking about the message how vividly did you imagine yourself doing something to increase the energy efficiency in your home,” and “How often did you imagine yourself doing something to increase the energy efficiency in your home.” Also, the items were slightly adapted to fit the message topic of the study; a mean score was calculated for environmental mental imagery (six items, Cronbach’s α = 0.83). For the items measuring c*ontent of environmental mental imagery* the ‘text’ item was adjusted. Participants were asked: “When you thought back to the message to what extent did you think about the text,” and the response scale ranged from 1 (Not at all), to 5 (I only thought about the text). Finally, the *pro-environmental goals* item was adjusted for the topic of the message: “When you thought back to the message to what extent did you think about what you could do in your day-to-day life to save energy.”

Next to these items, Study B included the following additional measures. Three items (Cronbach’s α = 0.66) were included to measure *pro-environmental behavior change* related to the heat loss message (e.g., Check that the windows are closed when the heating is on). Participants were asked to indicate whether there had been any change in their behavior in the past week (since seeing the message), on a scale ranging from 1 (Less) to 5 (More).

*Environmental values* were assessed using a 4-item scale (Cronbach’s α = 0.92; see **Table 1**) designed by De Groot and Steg (2008), based on Schwartz’s value scale (Schwartz, 1992). Participants were asked to rate the importance of four values as a guiding principle in their lives on a scale ranging from -1 (opposed to the values), 0 (not at all important), to 7 (of supreme importance): respecting the earth (harmony with other species); unity with nature (fitting into nature); protecting the environment (preserving nature); preventing pollution (protecting natural resources).

### Results

#### Experience of Environmental Mental Imagery

Similar to Study A, participants reported experiencing a moderate degree of environmental mental imagery (see **Table 1**). Again, thoughts about the visual content of the message were more frequent compared to the verbal content of the message, *t*(42) = 1.81, *p* = 0.077, *d* = 0.57. This was illustrated in the qualitative data; a content analysis found that most participants described mental images which referred to the images in the slideshow (*N* = 39). The remaining reported mental images were based on a combination of the text and the images from the slideshow. Overall, **Table 2** shows that the imagery descriptions had a similar structure as in Study A varying between word associations and short narrative statements.

In addition to replicating findings from Studies A, B explored the effect of individual and message characteristics on environmental mental imagery. Both research in environmental (e.g., Boomsma and Steg, 2014) and cognitive (May et al., 2004; Kavanagh et al., 2005) psychology suggests that a topic is more likely to be elaborated upon when it has affective meaning for the individual – this was reflected in the data. In Study B a measure of environmental values was included: individuals with strong environmental values see protection of the environment as an important general goal in life (De Groot and Steg, 2008). Individuals with strong environmental values reported more vivid environmental mental imagery after exposure to the energy saving message (*r* = 0.38, *p* = 0.013).

Message characteristics were explored in Study B by comparing the use of a vivid message to a less-vivid message. Message content can vary in its level of vividness. For example, videos are often seen as more vivid than still images. Vivid information has several properties (e.g., emotionally interesting, more effectively processed at encoding, greater imageability) that presumably give it a stronger impact on judgments (Nisbett and Ross, 1980; Taylor and Thompson, 1982; Chaiken et al., 1989). Importantly, vivid message content is linked to the retrieval of vivid mental imagery and a stronger impact on judgment (Smith and Shaffer, 2000; Bywaters et al., 2004). To increase vividness of a message new technological media can be used which attract attention by offering a more direct sensory experience compared to indirect informational messages (Midden et al., 2007). A technology that is particularly relevant in the context of energy consumption is thermal imagery. Thermal images visualize temperature differences; they have various applications and can be used as a method to visualize domestic energy saving opportunities (Pearson, 2011; Goodhew et al., 2015). A slideshow with thermal images was compared to a slideshow with the same information visualized using schematic images (see Appendix A). The vivid-thermal message (*M* = 5.75, *SD* = 2.08) appeared to evoke slightly more vivid environmental mental imagery compared to the schematic slideshow (*M* = 5.03, *SD* = 1.66), *t*(41) = 1.26, *p* = 0.215, *d* = 0.39, but the difference was not statistically significant (although it shows a medium effect size).

#### Relationship between Environmental Mental Imagery and Pro-environmental Goals and Behavior

As in Study A, a significant positive correlation was found between pro-environmental thoughts and environmental mental imagery. In addition, participants who reported more vivid environmental mental imagery also reported more behavior change (see **Table 3**).

## Study C

### Materials and Methods

#### Participants and Procedure

In Study C, 78 university students (13 men, mean age = 20.9, *SD* = 4.5) were recruited via the university participant pool in return for course credit. In a lab study participants were shown a slideshow (include images and spoken text) depicting a future climate scenario. There were two conditions: *N* = 40 received a positive future scenario; *N* = 38 received a negative future scenario, further described in Section “Experience of Environmental Mental Imagery” and Appendix A (full materials are available online, doi: 10.13140/RG.2.2.12718.92484). Directly after the slideshow, all participants filled in a survey measuring pro-environmental goals. Participants returned to the lab 1 week later to fill in a follow-up survey. The consent and debrief procedure was the same as Study B.

#### Measures

Study C included the same measures as Study B with minor adjustments. The *Environmental Mental Imagery* scale was adjusted to fit the message topic (e.g., How vividly did you imagine problems related to sustainability); a mean score was calculated for environmental mental imagery (six items, Cronbach’s α = 0.88). *Content of environmental mental imagery* was assessed again, only the ‘text’ item referred to the ‘spoken text’ in Study C. In addition to the *pro-environmental goals* item included in Studies A and B (adjusted to the message topic of the study), Study C included a measure of pro-environmental goal intentions directly after exposure to the message. Participants were asked to indicate their intentions for the future on a scale ranging from 1 (Probably not) to 7 (Yes, definitely). The scale consisted of seven items (Cronbach’s α = 0.84) measuring difficult intentions (e.g., Actively volunteer for an environmental organization; Completely change my lifestyle to live fully sustainably), and eight items (Cronbach’s α = 0.64) measuring easy intentions (e.g., Only boil the water I need in the kettle; Take my own bags to the shops). Nine items (Cronbach’s α = 0.79) were included to measure general *pro-environmental behavior change* (e.g., Take my own bags to the shops), using the same response scale as Study B. Finally, the scale from Study B was used to assess environmental values (Cronbach’s α = 0.85; see **Table 1**).

### Results

#### Experience of Environmental Mental Imagery

The results from Studies A and B were replicated: participants reported experiencing environmental mental imagery around the mid-point of the scale (**Table 1**). Furthermore, participants mainly thought about the visual content of the message compared to the verbal content of the message, *t*(77) = 2.02, *p* = 0.047, *d* = 0.23, as confirmed by the qualitative data: most mental images referred to images from the slideshow (*N* = 51). The remaining image descriptions were based on a combination of images and text from the slideshow or text alone. These image descriptions took the form of simple word associations and short narrative statements (see **Table 2**). Lastly, respondents with strong environmental values were more likely to experience vivid environmental mental imagery in response to the sustainability message, *r* = 0.45, *p* < 0.001.

Study C further explored the role of message characteristics. Study A exposed individuals to a negative message and Study B used a neutral message. So, to examine whether mental imagery is also evoked when a positive visual message is used, about half of the participants in Study C was exposed to a slideshow depicting a positive future scenario indicating what the future could be like if people changed their behavior. The other half of the participants was exposed to a negative future scenario indicating what the future could be like if people did not change their behavior (see Appendix A). The results showed that the negative future scenario (*M* = 5.11, *SD* = 2.10) led to equally vivid environmental mental imagery as the positive future scenario (*M* = 5.38, *SD* = 1.92), *F*(1,76) = 0.35, *p* = 0.555, η_p_^2^ = 0.01.

#### Relationship between Environmental Mental Imagery and Pro-environmental Goals and Behavior

The positive correlation between environmental mental imagery and pro-environmental thoughts was found again, as well as the positive relationship between environmental mental imagery and self-reported behavior change (**Table 3**). Study C extended these findings by including a more elaborate measure of pro-environmental goals in the form of goal intentions. Environmental mental imagery was shown to associate more strongly with intentions for difficult behaviors compared to easy behaviors (**Table 3**).

**FIGURE 1:**
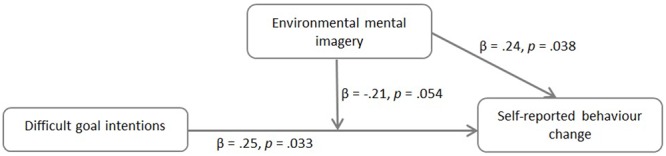
**Moderation effect between pro-environmental goals for difficult behavior, environmental mental imagery and self-reported behavior change.** *R*^2^ change after adding interaction effect to the model, *F*(1,72) = 3.85, *p* = 0.054.

Based on the cognitive literature suggesting that mental imagery can help make a goal more focal and increase its influence on behavior, we explored whether environmental mental imagery about the message can increase the motivational power of pro-environmental goals, using a moderation analysis. So, we examined whether the relationship between pro-environmental goals and self-reported behavior change was influenced by the experience of environmental mental imagery. This analysis could only be conducted for Study C where pro-environmental goals were measured directly after the message before the measure of mental imagery 1 week after seeing the measure. A tendency was found that environmental mental imagery moderated the relationship between intentions for difficult behavior and self-reported behavior change (see **Figure 1**). That is, the variance explained by the predictors increased marginally (from *R^2^* = 0.17 to *R^2^*= 0.21) after the interaction between intentions for difficult behavior and environmental mental imagery was added to the model of intentions for difficult behavior and imagery alone, *F*(1,72) = 3.85, *p* = 0.054. The same relationship was not found for intentions for easy behavior, *R^2^* change *F*(1,74) = 0.18, *p* = 0.676.

**FIGURE 2:**
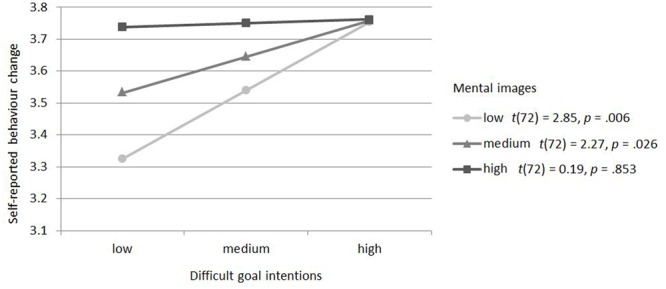
**Relationship between difficult pro-environmental goals and self-reported behavior change for low, medium and high environmental mental imagery**.

**Figure 2** was created on the basis of the regression equation; it depicts the relationship between difficult pro-environmental goal intentions and behavior change for low (one standard deviation below mean), medium (mean), and high (one standard deviation above mean) environmental mental imagery. The data suggest that there may be some support for a relationship where participants with weak pro-environmental goals for difficult behaviors reported behavior change similar to participants with strong pro-environmental goals, if they experienced vivid environmental mental imagery. In the case of less vivid environmental mental imagery, behavior change for participants with weak pro-environmental goals for difficult behaviors appeared to be lower than for participants with strong pro-environmental goals. A simple slopes analysis depicted in **Figure 2** found support for these trends in the data. If participants experienced vivid environmental mental imagery there was no significant difference in self-reported behavior change for participants with weak and strong pro-environmental goals for difficult behaviors (*p* = 0.853). If participants experienced medium or low environmental mental imagery, self-reported behavior change was lower when participants had weak compared to strong pro-environmental goals for difficult behaviors (*p* = 0.026; *p* = 0.006, respectively).

Thus, the results provide some support for the idea that vivid environmental mental images help strengthen the effect of weak pro-environmental goals on behavior, or make existing goals more focal – but it should be noted that this effect was only marginally significant.

## General Discussion

The results from the three studies show that visual environmental messages are internalized to form environmental mental images with a moderate degree of vividness. The recall of the messages was mostly visual in nature but there were individual differences in the structure of the environmental mental images experienced by participants, ranging from one or two word associations to elaborative statements. Consistent and strong associations were found between mental images and the formation of pro-environmental goals and self-reported behavior change. Environmental mental imagery was also found to relate more strongly to goal intentions for difficult behaviors rather than easy behaviors. These are behaviors that are particularly important in reducing the impact of environmental change. This finding therefore emphasizes the importance of studying the role of environmental mental imagery in motivating pro-environmental behavior. In addition, some support was found for a moderating role of mental imagery: strengthening the motivational power of weak pro-environmental goals for difficult actions.

These initial studies focused on examining the role of environmental mental imagery in communicating pro-environmental messages, and at this stage the aim was not to pinpoint the circumstances under which visual messages would be most effective in motivating pro-environmental behavior. But we can already identify some factors that could influence whether a message is able to trigger recurring and vivid environmental mental imagery. Studies B and C showed that respondents with strong environmental values, for whom an environmental message will be especially important, reported experiencing more vivid environmental mental imagery after seeing the message. Furthermore, Study C showed that vivid environmental mental imagery can be evoked after exposure to messages using positive and negative emotions. The effect of message vividness, explored in Study B, was inconclusive and is a topic that warrants further investigation.

Overall, the current studies provide insight into the type of information that environmental mental imagery provides. For instance, the goal to be more careful with plastic waste might be reflected by environmental mental images depicting the negative consequences of plastic on wildlife. At the same time, less vivid environmental mental images on this topic reflect a weaker goal to reduce plastic waste. There are some limitations that need to be acknowledged. First, the sample sizes were relatively small and consisted mainly of university students. This might limit the generalizability of these results to wider populations, as younger age is associated with pro-environmental values, and environmental skepticism is less common in individuals with higher levels of education (Whitmarsh, 2011). Second, the studies relied strongly of self-report. But, these studies are meant to be a first step in a research program introduced in the next section where solutions for these limitations will be addressed.

## Program of Research

This literature review and the initial findings described above pose a number of questions that should be investigated further. The review of the literature showed that visual information is widely used to communicate environmental change and, though it is assumed that images are internalized, little is known about how such mental imagery influences pro-environmental goals and behavior. Cognitive psychological research suggests that mental imagery is tightly linked to emotion and goals, so we are more motivated to do things that we imagine vividly. We presented three preliminary studies that showed that recall of environmental change messages often takes the form of mental imagery rather than verbal recall, that this mental imagery may be elaborated to encompass imagery of what the individual can do themselves to prevent the problem, and that mental imagery is positively associated with intentions to change behavior and self-reported actual change. The following program of research would explore key questions that remain: how does environmental mental imagery influence pro-environmental behavior? Which individuals factors influence whether vivid environmental mental images are formed, and what sort of messages are likely to have the greatest impact via imagery? Can manipulations of environmental mental imagery strengthen the influence of a message on pro-environmental behavior? In this section these potential areas of future research are discussed.

### Environmental Mental Imagery as a Trigger and Motivator

Two motivational roles of mental imagery were first proposed in Section “Applying a Cognitive Framework to Environmental Mental Imagery.” Based on the cognitive literature it was suggested that: (1) mental images can increase the motivational power of long-term sustainable goals, and (2) affectively charged external images of environmental change can become internalized so they can serve as triggers for environmental behaviors. Initial tentative support was found in Study C for mental imagery strengthening goals. We suggested that environmental mental imagery about a pro-environmental message could act as an amplifier, strengthening the relationship between pro-environmental goals and behavior change. In other words, mental images might provide the medium by which goals are converted into behaviors. In Study C, some indication was found for a moderation effect for difficult pro-environmental behaviors, but more research is needed to confirm these findings. The second motivational role, that mental imagery can trigger behaviors, could be examined by conducting a mediation analysis. In this case environmental mental images about the pro-environmental message are expected to trigger pro-environmental goals which in turn can increase the likelihood that individuals will act more pro-environmentally. Thus, environmental mental images are expected to remind individuals about the benefits of behaving according to long-term environmental goals when there is not necessarily an external (visual) reminder available.

### Exploring the Influence of Individual and Message Characteristics on Environmental Mental Imagery

Factors that enhance the ability to imagine or rehearse imagery should strengthen motivation. Individual characteristics and message characteristics may play a role here. Lorenzoni et al. (2007) state that individuals differ in terms of personal beliefs, knowledge, values, experience, social network and demographic background. This can lead them to perceive environmental change and barriers to engagement differently. So, when seeing a visual message people will interpret this in different ways: a certain image may lead to change in one individual, while the same does not happen in another, depending on the characteristics of the individual. This is reflected in research on mental imagery. Whether an initial goal-related thought is elaborated upon depends on whether the topic has affective meaning for the individual (May et al., 2004; Kavanagh et al., 2005). The role of values was briefly discussed in the current studies: strong environmental values were related to the experience of vivid environmental mental imagery. But, further in-depth exploration on the relationship between individual characteristics and environmental mental imagery is needed. One individual factor that could also be particularly relevant in this regard is ‘imagery ability’. Individuals might differ in their spontaneous use of imagery in day-to-day life, with some experiencing vivid and picture-like visual imagery while others rely less on visual imagery (cf. Reisberg et al., 2003; Andrade et al., 2014).

With regard to message characteristics there are a number of factors that might influence whether a message leads to recurring vivid environmental mental imagery. We have started to explore some of these factors in the studies above, but these require further investigation. There is a lot of debate around the influence on message vividness, and the effect itself might not be robust (Taylor and Thompson, 1982). But there is reason to believe that vivid message content is linked to the retrieval of vivid mental imagery and a stronger impact on judgment (Smith and Shaffer, 2000; Bywaters et al., 2004). Study B only found a weak effect of message vividness, however, two messages were compared that were relatively close in vividness (e.g., both were visual in nature). A stronger effect on environmental mental imagery would be expected when using messages on either end of the vividness spectrum.

There tends to be agreement in the literature that part of the motivational power of images comes from their ability to evoke emotions (cf. Sheppard, 2005). Using affectively charged images is therefore thought to be an effective method ‘to lure people in’ and motivate behavior change (Joffe, 2008; Andrade et al., 2012). Nature-based imagery (especially including water) has been found to have a strong impact on affect (c.f. White et al., 2010). Moreover, the effect of emotions on behavior has been supported by neuroimaging research: strong emotive responses to messages on environmental degradation can predict willingness to engage in conservation behavior (Sawe and Knutson, 2015). However, there is debate on the type of emotions that are most effective in influencing judgements (e.g., on the use of negative frames, see Ruiter et al., 2001; O’Neill and Nicholson-Cole, 2009; Moser, 2010; Spence and Pidgeon, 2010; Morton et al., 2011). An interesting question relates to which emotional frames are most likely to lead to vivid environmental mental imagery. Study C showed that both negative and positive messages can evoke similarly vivid imagery. Future research could explore this further by examining environmental mental imagery content when individuals have been exposed to a mixed emotional content (e.g., positive and negative imagery). Also, environmental mental imagery in response to other types of emotional framing such as disgust (e.g., Joffe, 2008) and guilt (e.g., Pelletier et al., 1998) could be explored. Studies that follow individuals over longer time periods are needed to test if positive and negative messages have different long-term impact. It is conceivable that positive imagery, of beautiful, clean, green futures, will be rehearsed more often because rehearsal reinstates the positive emotion and is therefore rewarding in itself, whereas fear appeals might have initial ‘shock value’ they may be rehearsed less often (Kavanagh et al., 2005; Andrade et al., 2012).

Message tailoring is another message characteristic which could influence the extent to which a topic has affective meaning for an individual, and thus whether vivid mental images are formed and elaborated upon. Tailored, or personalized, information can help individuals in making the link between their behavior and specific environmental consequences. This type of information is often lacking in the public domain (Thaler and Sunstein, 2009). A recent study on the use of thermal images to encourage energy efficiency behaviors has suggested that tailored visuals could lead to more intrusive mental images compared to non-tailored visuals. Householders who received tailored thermal images (of their own home), reported that these popped into mind more frequently in the weeks after seeing the images, compared to householders who received non-tailored thermal images (of other people’s homes; Boomsma et al., 2016).

A final point here relates to the use of textual information. In the public domain, individuals often receive information with a mix of visuals, texts and sounds – which makes it difficult to study the effect of visuals and text in isolation (Joffe, 2008). But, the effect of textual information should also depend on whether it is imaginable. Recent studies even suggest that more vivid images might be formed if individuals are given the opportunity to construct their own images. In a study by Krans et al. (2010) participants were instructed to form mental images based on a verbal report describing the aftermath of a road traffic accident. The emotional impact of forming one’s own images was higher compared to the emotional impact reported by participants who viewed the original film on which the verbal report was based. A similar study could be conducted in the context of environmental change to test whether images constructed from a verbal report can have a stronger impact compared to images constructed from external visual images.

### Direct Manipulations of Mental Imagery

The research presented so far has been mainly correlational in nature. Experimental manipulations (e.g., using cognitive research methods) of imagery should provide stronger tests of the importance of mental imagery for pro-environmental behaviors. Mental imagery requires activation of working memory – a part of the memory system which enables an individual to consciously keep information in mind, transform this information, or use it to achieve a goal (Andrade et al., 2012). The Working Memory model (Baddeley and Hitch, 1974; Baddeley et al., 2011) assumes that working memory consists of four limited-capacity components, including the visuospatial sketchpad, which temporarily maintains and manipulates visual information and is important for vivid visual mental imagery (Baddeley and Andrade, 2000). A second temporary storage system is the phonological loop, which performs a similar function with auditory and verbal information.

Because the visuospatial sketchpad and phonological loop have limited processing capacity, performing a visuospatial or verbal task reduces ability to temporarily maintain and manipulate other representations in the same sensory modality simultaneously. In a study by Baddeley and Andrade (2000) participants were asked to form a mental image of an external image. A concurrent simple visuospatial task (e.g., tapping a pattern on a keyboard), reduced the vividness of visual mental images more than did a concurrent verbal task (e.g., counting aloud); the converse pattern of results was obtained for auditory mental imagery. Research on trauma memories has shown that competing visuospatial tasks not only reduce the vividness of visual recollections but also weaken the emotions (e.g., distress) felt when recollecting the traumatic event (Lilley et al., 2009). Similar findings are obtained for other emotive autobiographical memories (e.g., Andrade et al., 1997; Kavanagh et al., 2001; Van den Hout et al., 2001). Visuospatial tasks completed during, or immediately after, exposure to a traumatic event also reduce the likelihood that distressing images will intrude into awareness in subsequent days (Holmes et al., 2004; Stuart et al., 2006; Holmes and Bourne, 2008; Krans et al., 2010). This effect on intrusiveness of the image is important because goal-related images will only motivate behavior if they are easily triggered by cues that signal opportunity to engage in the behavior (Kavanagh et al., 2005; Andrade et al., 2012).

In the context of environmental mental imagery, we predict that tasks that interfere with the generation and rehearsal of images will reduce the impact of an environmental message on pro-environmental intentions and behavior. We hypothesize that the converse will also be true. For instance, there is preliminary evidence that developing vivid goal-related images and training rehearsal of those images, using a novel counseling technique called Functional Imagery Training (Kavanagh et al., 2014), can strengthen motivation and goal achievement. One area of research where this approach might be particularly relevant is the promotion of sustainable eating habits (e.g., reducing meat consumption, buying local food). Research has shown that through practicing positive goal imagery individuals can change their eating behaviors toward a healthier lifestyle (Andrade et al., 2016) – a similar approach could be used to work toward a more sustainable lifestyle.

So, we predict that interventions, individual differences or message characteristics that increase vividness, availability and elaboration of environmental mental imagery will increase the impact of an environmental message on behavior.

### Environmental Mental Imagery: Other Modalities?

In general mental images occur as sensory images and can be visual, auditory, olfactory, taste, touch, bodily sensations, and emotional feelings (Andrade et al., 2014). The same is expected for environmental mental imagery. For example, individuals might experience olfactory imagery (e.g., smelling the sea when thinking back to a marine pollution message), or emotional imagery (e.g., imagining the sad feelings that the marine pollution message evoked). Taking together the results of some of the studies examining environmental mental imagery in the public (e.g., Leiserowitz, 2006; Smith and Leiserowitz, 2012), it seems that the reported imagery is mostly visual in nature – with associated affect. This might reflect the type of information available in the public domain. We predict that messages that elicit vivid multisensory imagery may more strongly motivate pro-environmental behavior. Research could look into designing a scale, or adapting an existing scale, to measure these other types of mental imagery with respect to environmental messages. Using this scale, different types of messages could be compared in their ability to elicit multisensory imagery and motivate pro-environmental behavior. A second line of research could look into designing messages specifically targeting certain sensory images and comparing their effect on mental imagery vividness and pro-environmental behavior.

### Recognizing Research Context

In this review research from various disciplines has been discussed, all using different methods to study and measure mental imagery (e.g., representative surveys in Leiserowitz, 2006, interviews in Nicholson-Cole, 2005, and experimental approaches in May et al., 2004). Two points can be taken from these approaches. First, it is worth emphasizing the benefits of a mixed method approach using both qualitative and quantitative methods to capture rich data that allows us to assess both the vividness and content of environmental mental imagery. Second, although a lab setting may be appropriate (and perhaps necessary) to examine the cognitive mechanisms underpinning the effect of mental imagery of pro-environmental goals and behavior, the applied nature of this research also calls for field studies that acknowledge the context in which environmental mental imagery is formed. Also, a field setting could enable a more accurate observation of pro-environmental behavior and its relationship with environmental mental imagery.

## Conclusion

As noted by Smith and Leiserowitz (2012, p. 1030), mental images ‘are individual mental representations and feelings [that] cannot be separated from larger-scale political, economic and cultural dynamics.’ Different actors within our social systems influence the mental images that we have of environmental change. Environmental imagery available in the public domain, internalized as mental images, can have an important influence on the pro-environmental goals, and indeed, mitigation strategies that individuals adopt. This review calls for an increased awareness of the visual information currently used in the public domain to communicate environmental change. Messages which evoke vivid environmental mental images have the potential to strengthen existing and newly developed pro-environmental goals which can bring individuals one step closer to a more sustainable lifestyle.

## Author Contributions

All authors contributed to the development of the theoretical framework. CB drafted the manuscript, developed the research questions, collected the data, and interpreted the results. SP and JA provided comments on the manuscript. All authors approved the final version of the manuscript for submission.

## Conflict of Interest Statement

The authors declare that the research was conducted in the absence of any commercial or financial relationships that could be construed as a potential conflict of interest.
