# Supplementary material for: Imagining Change: An Integrative Approach toward Explaining the Motivational Role of Mental Imagery in Pro-environmental Behavior
Source: Front Psychol. 2016 Nov 17;7:1780. doi: 10.3389/fpsyg.2016.01780 (PMC5112244; doi:10.3389/fpsyg.2016.01780)
Supplement: Supplementary file 1 [file Image_1.pdf]

Appendix A: Messages used in Studies A, B and C

Study A: Great Pacific Garbage Patch Video
The video consists of a lecture by Captain Charles Moore about plastic pollution in the ocean (TED, 2009). The video depicts Charles Moore talking in front of an audience interspersed by images from a slideshow. In total the video is 7 minutes and 23 seconds long, more than half of the time images are displayed on screen (4 minutes, 30 seconds). Additionally the final 40 seconds of the video consists of a news report including various images. Some examples of images used in the video can be found in Box A1 below.

Study B: Energy Saving Message
The energy saving message consisted of a slideshow with text and images about energy saving measures. Before the start on the slideshow participants were shown three slides with instructions on how to read the images. After the instructions participants in both conditions were exposed to six slides with an image, either thermal or schematic depending on the condition. The text was exactly the same in both conditions. The slideshow was shown on a computer screen, while participants were seated alone in the lab. See Box A2 for examples of the images and text used in each slideshow.

Study C: Future Scenarios
Before the start of each slideshow participants saw a slide with the text: “This is a slideshow depicting a prediction of a realistic possible future scenario that could occur in 50 years time”. Both slideshows consisted of 13 slides with 20 images and spoken text. The negative future slideshow was four minutes and 37 seconds long, and the positive future slideshow was four minutes and 22 seconds long. Thus, in both conditions the average time per slide was approximately 25 seconds. The slideshow was shown on a computer screen, while participants were seated alone in the lab. Examples of the images and text used in the negative (Box A3a) and the positive slideshow (Box A3b) are depicted below.

Box A1: Study A - Example images included in GPGP Video (images courtesy of TED, 2009)

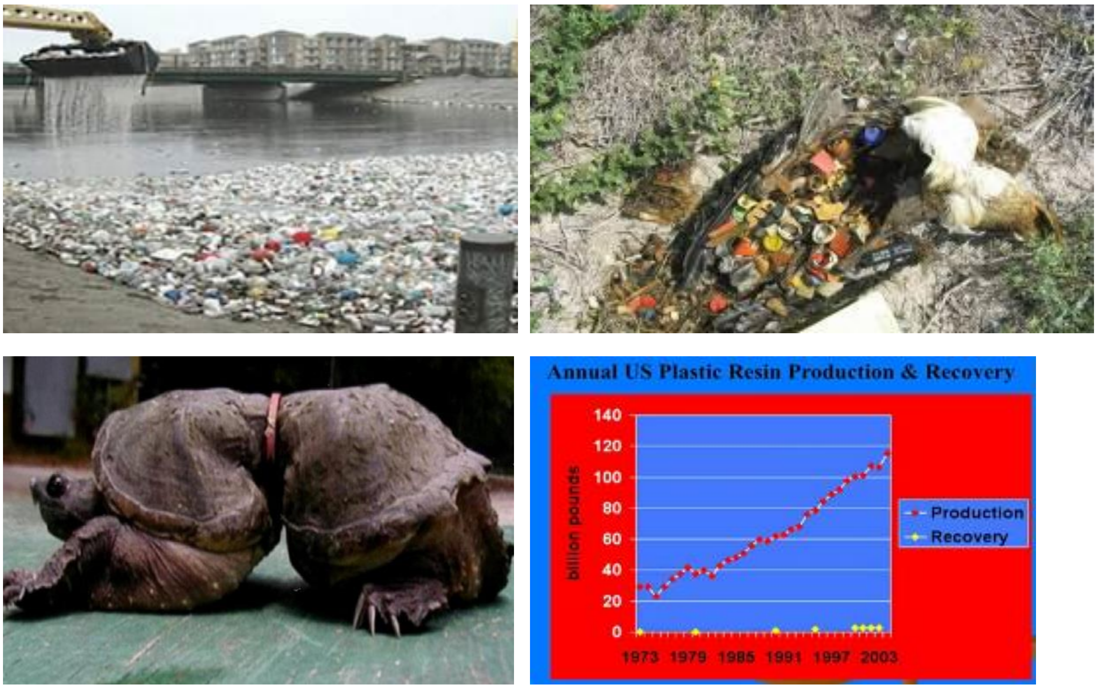

Box A2: Study B - Images and Text included in the Energy Saving Message (images courtesy of Goodhew, 2013)

<table>
<tr><td>Thermal Image</td><td>Schematic Image</td></tr>
<tr><td>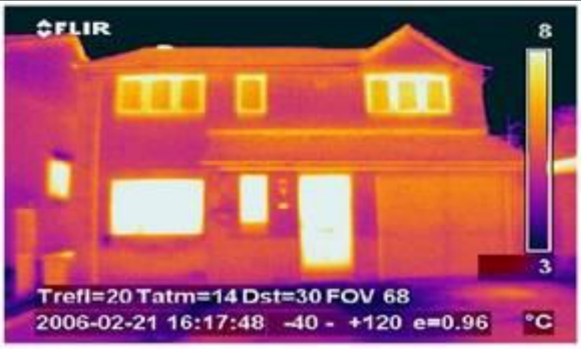
</td><td>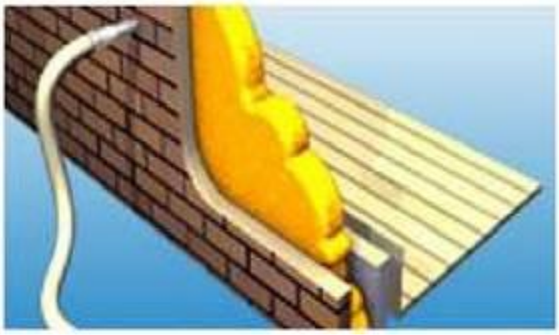</td></tr>
<tr><td colspan="2">Text: Home insulation - Because around half of heat loss in a typical home is through the walls and loft, it’s worth checking whether yours are insulated.</td></tr>
<tr><td>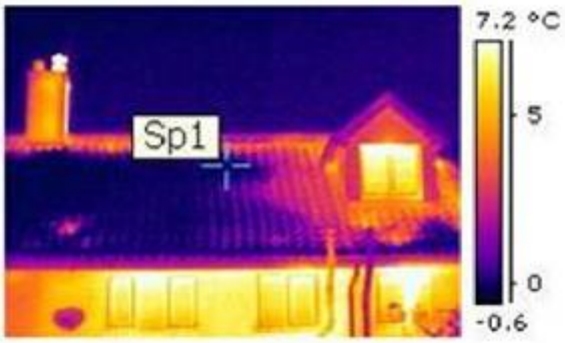
</td><td>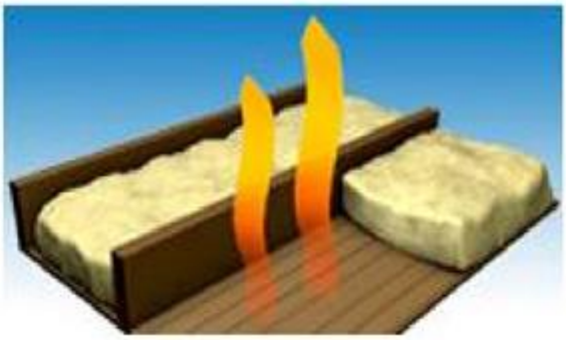</td></tr>
<tr><td colspan="2">Text: Loft insulation - Insulating your loft could save you around £150 per year on your energy bills if you don’t have any insulation there at the moment. If everyone in the country topped up their loft insulation to 270mm, the UK would save around £520 million each year!</td></tr>
<tr><td>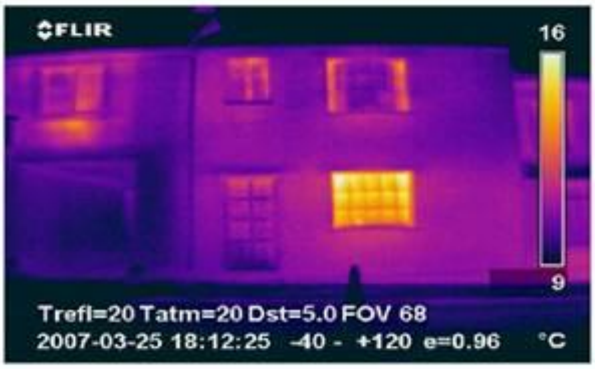
</td><td>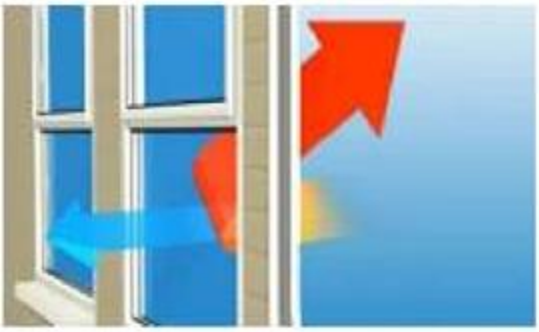</td></tr>
<tr><td colspan="2">Text: Glazing - Double glazing cuts heat lost through windows by half and installing Energy Saving Recommended double glazing could save around £135 a year on your heating bills. Double glazing works by trapping air between two panes of glass creating an insulating barrier that reduces heat loss, noise and condensation.</td></tr>
</table>

Box A3a: Study C - Examples of Images and Text included in the Negative Future Scenario (images courtesy of Jenkin et al., 2010)

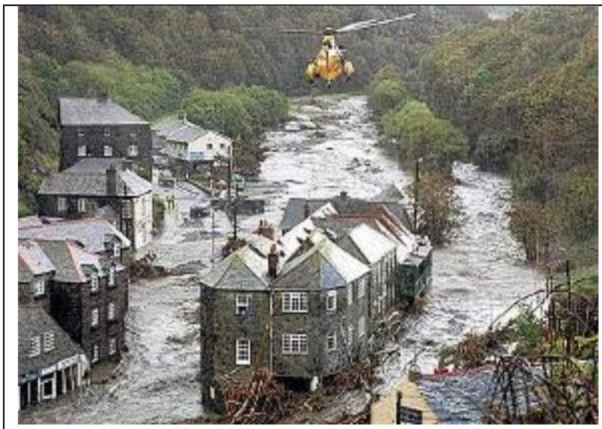

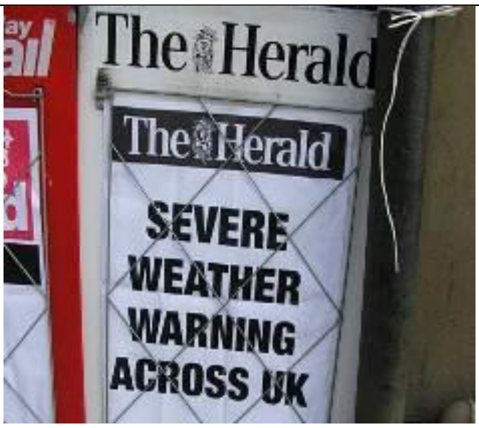

Spoken text: It is the crises that drive sustainable development. Many regions have experienced water shortages, floods and droughts, storms and erosion, sea-level rise, toxic accumulation, food chain risks, genetic disruption, radiological contamination, morbidity and tropical diseases, and ecological and agricultural stress.

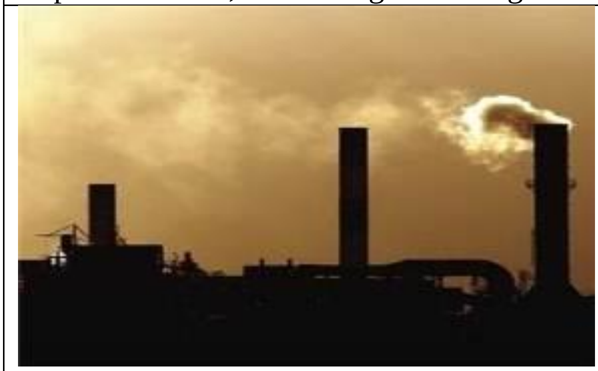

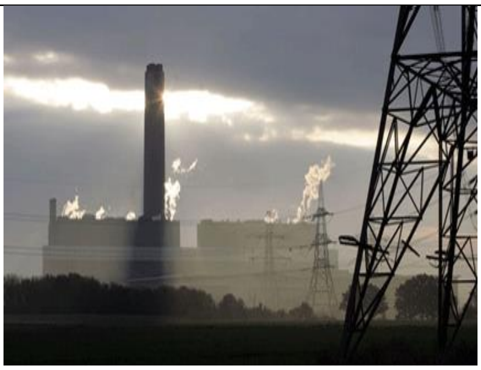

Spoken text: Energy prices are high as this is proving the only way to reduce demand for the diminishing resource. The energy sector is based on plentiful supply of fossil fuels. There is a strong tendency to preserve existing sources of energy including native coal and nuclear power by extending the lives of existing stations.

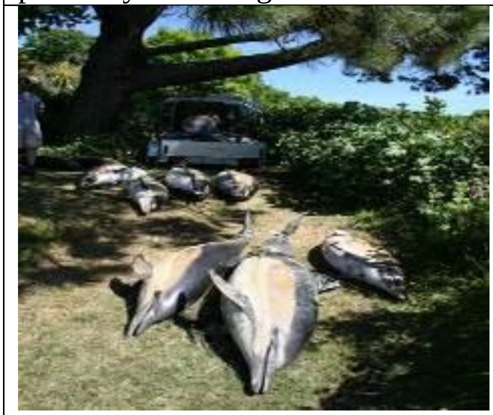

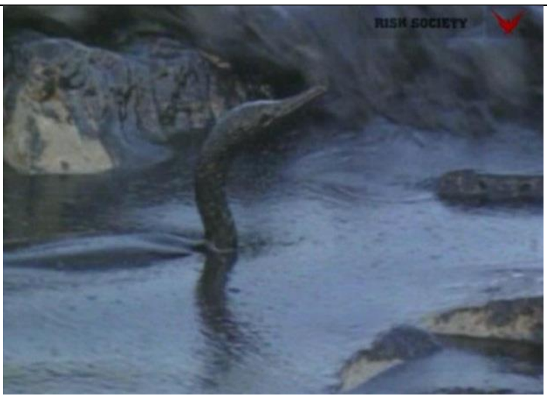

Spoken text: Environmental pollution and the depletion of water resources put stress on animal and plant species.

Box A3b: Study C - Images and Text included in Positive Future Scenario (images courtesy of Jenkin et al., 2010)

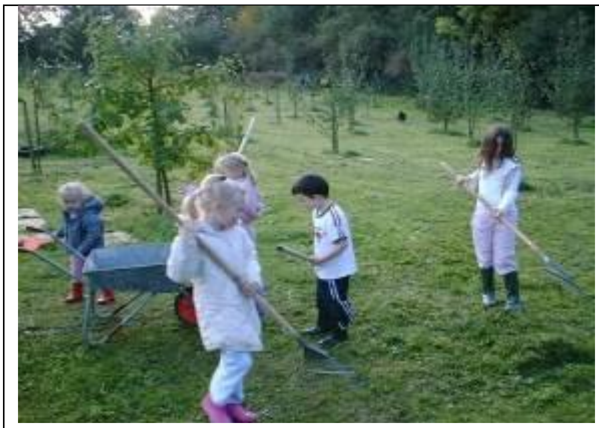 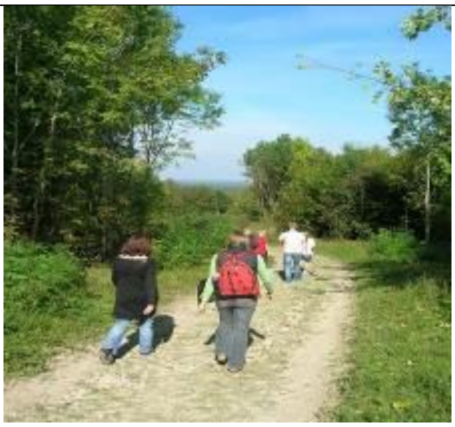

Spoken text: Social values are community-oriented, encouraging cooperative self-reliance and regional development

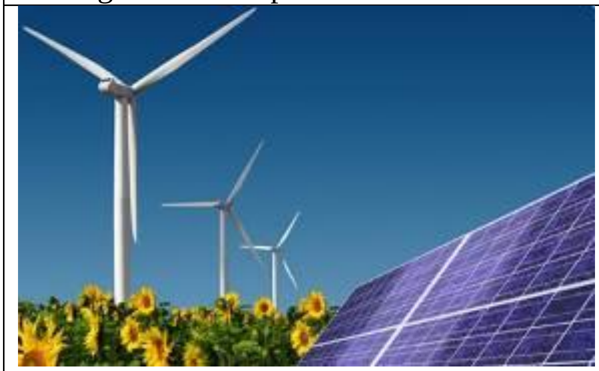 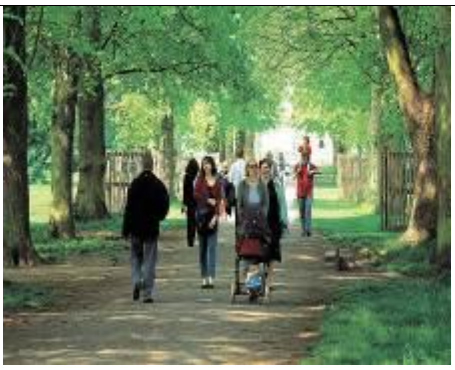

Spoken text: Economic growth is not an absolute political priority. Instead, there is a strong emphasis on equity, social inclusion and democratic values. The conservation of resources and the protection of the natural environment are strong political objectives.

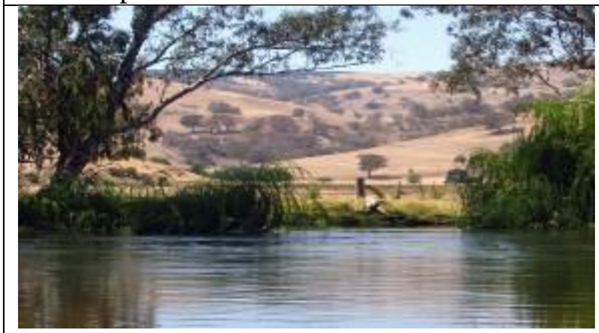 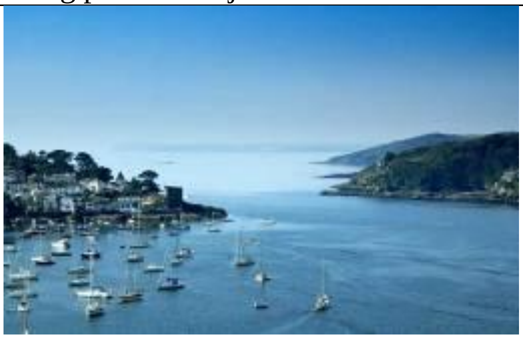

Spoken text: Water demand falls. Consumers install water conservation techniques, grey water systems and radically reduce the use of public supply water in gardens. There is an increasing consciousness that water resources have to be protected. Water quality improves dramatically as a result of acute concerns about the quality of the local environment, reduced pesticide use and changes in industrial structure.

References

Goodhew, J. (2013). *Making Heat Visible: Improving household heat efficiency through thermal images* (Doctoral thesis, University of Plymouth, United Kingdom). Retrieved from https://pearl.plymouth.ac.uk/handle/10026.1/1956

Jenkin, R., Walter, J.L., & Pahl, S. (2010). *Inducing fear or raise hope.* University of Plymouth, Unpublished data.

TED. (2009). Captain Charles Moore on the seas of plastic. Retrieved from http://www.ted.com/talks/capt_charles_moore_on_the_seas_of_plastic.html.
